# Magnetic-Plasmonic Heterodimer Nanoparticles: Designing Contemporarily Features for Emerging Biomedical Diagnosis and Treatments

**DOI:** 10.3390/nano9010097

**Published:** 2019-01-13

**Authors:** S. Fatemeh Shams, Mohammad Reza Ghazanfari, Carolin Schmitz-Antoniak

**Affiliations:** 1 Peter-Grünberg-Institut (PGI-6), Forschungszentrum Jülich, 52425 Jülich, Germany; c.schmitz-antoniak@fz-juelich.de; 2 Department of Materials Science and Engineering, Ferdowsi University of Mashhad, Mashhad 9177948974, Iran; ghazanfari.mr@gmail.com

**Keywords:** hybrid nanostructures, interfacial morphology, electronic structure, biocompatibility, noble metals, magnetic ferrites, hyperthermia, photothermal therapy, bioimaging, drug delivery

## Abstract

Magnetic-plasmonic heterodimer nanostructures synergistically present excellent magnetic and plasmonic characteristics in a unique platform as a multipurpose medium for recently invented biomedical applications, such as magnetic hyperthermia, photothermal therapy, drug delivery, bioimaging, and biosensing. In this review, we briefly outline the less-known aspects of heterodimers, including electronic composition, interfacial morphology, critical properties, and present concrete examples of recent progress in synthesis and applications. With a focus on emerging features and performance of heterodimers in biomedical applications, this review provides a comprehensive perspective of novel achievements and suggests a fruitful framework for future research.

## 1. Introduction

Aqueous colloids of heterodimer nanoparticles are a fascinating class of multimodal hybrid structures, which have appeared in the last decade and increasingly attracted many attentions. In general, heterodimer structures are composed by targeted and controlled junction of two main parts as a guest/host template with simultaneous retention of the key properties of components as well as the influence on the conjugated part through interfacial interactions.

In most cases, magnetic nanoparticles have been firstly pre-synthesized via common methods and employed as host seeds for the chemical deposition of the plasmonic component [1,2], so that the crystallized magnetic-plasmonic heterodimers consist of magnetic hosts and plasmonic guests. Although heterodimer nanostructures have been commonly synthesized based on the same protocols of seed-mediated process [1,2,3], utilization of various techniques, such as coprecipitation [4,5,6,7,8,9], thermal decomposition [10,11,12,13,14], solvothermal [15,16], flame aerosol [17,18], and Brust method are reported [19,20,21].

The elaborate design of heterodimer nanoparticles does not only result in a combination of the critical characteristics of both components in a single structure, but also leads to emerging new excellent features [22,23,24,25]. The resulting heterodimer can be greater than the sum of its parts because of mutual influences of the constituents leading to new characteristics e.g., by structural modifications, electronic hybridization, or exchange coupling phenomena at the interface. Particularly, the combination of metallic/nonmetallic nano-ingredients with different magnetic, electronic, optical, and chemical properties lead to impressive developments in a wide range of research fields, ranging from basic research perspectives to catalytic [26,27,28,29,30,31,32,33,34], magnetic [35,36], electronic [37], and biomedical [38,39,40,41,42,43] applications.

**Scheme 1:**
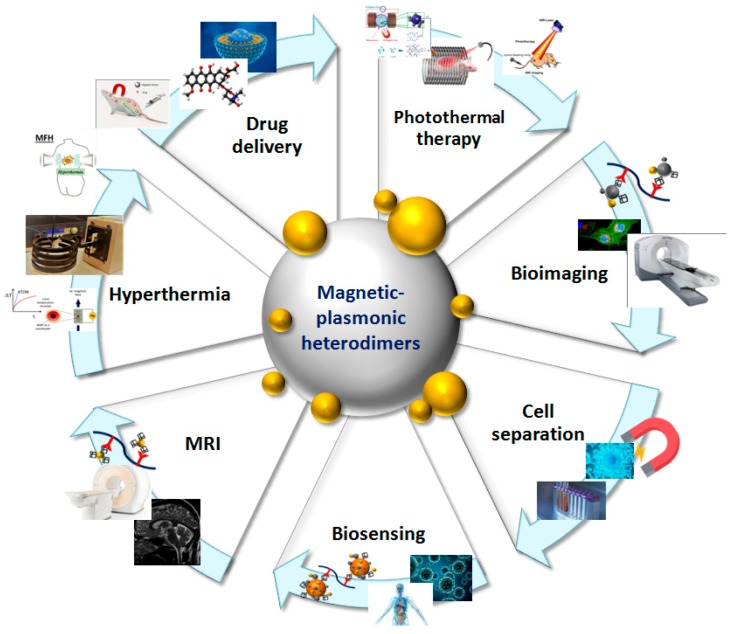
Potential biomedical applications of magnetic-plasmonic heterodimers.

The latter was pioneered in 2005 by Yu et al. who designed novel Fe_3_O_4_-Au nano-heterodimers [44,45]. Subsequently, the positive roles of different magnetic-plasmonic heterodimers for various biomedical diagnostic and therapeutic treatments have been reported, including magnetic resonance imaging (MRI) [46,47,48], cellular uptake [49,50,51], plasmon imaging [52,53], X-ray computed tomography (CT) [54], positron emission tomography (PET) [55], photothermal therapy (PTT) [56,57,58], magnetic fluid hyperthermia (MFH) [59,60], and drug/gene delivery [1,61] (Scheme 1). More general, the magnetic component provides potential features, like magnetic targeting of structure and heat-generation, while the plasmonic constituent is responsible for photothermal phenomena, plasmonic imaging, cell tracking, and improvement of biocompatibility.

In order to reveal hitherto unconsidered aspects of hybrid nanomaterials, our review article is focused on recently reported works about the design of magnetic-plasmonic heterodimer nanostructures for biomedical applications. To this end, we firstly identify the important desired magnetic and plasmonic properties for different applications in Section 2. In Section 3, different structural designs are presented for the creation of novel magnetic-plasmonic heterodimers and are categorized according to their electronic nature (metallic vs. nonmetallic) and interfacial morphologies. In Section 4, selected examples of successfully tailored magnetic, plasmonic, electronic, and biocompatibility properties of heterodimers are summarized before briefly addressing improved performances in associated applications in theranostics in Section 5. Finally, in Section 6, an outlook on possible future developments in the mentioned fields is presented.

## 2. Hot Properties for Biomedical Applications

Before turning to the discussion of recent developments in tailoring heterodimers for biomedical applications, a brief summary of magnetic and plasmonic characteristics shall be given here, which are desired in addition to biocompatibility. Exemplarily, hyperthermia cancer treatment, MRI, and magnetically targeted drug delivery have been chosen for the magnetic part, and photothermal therapy and bioimaging were chosen for the plasmonic part.

### 2.1. Magnetic Characteristics

The first requirement for magnetic particles in any biomedical application is superparamagnetism, i.e., a vanishing remanent magnetization that is caused by thermal fluctuations of the magnetization direction. Otherwise, the particles will form large magnetic agglomerates, yielding unwanted side effects, like thrombosis.

Magnetic hyperthermia is one the novel methods for cancer therapy that results in destroying cancerous cells during localized temperature rising up to around 43 °C upon applying AC magnetic field with minimal side effects on the normal cells [62]. In fact, the biological integrity of tumor cells membrane and their cytoskeleton are damaged during hyperthermia [63], which is firstly introduced using Fe_2_O_3_ nanoparticles in 1957 [64]. In this method, heating efficiency is closely related to the external AC magnetic field amplitude and frequency, as well as magnetic nanoparticles characteristics, such as anisotropy, magnetization, inter-/intra-particles interactions, particles size, and size distribution [62,63,64,65,66]. The conversion of magnetic work to internal energy has been described by Rosensweig [65] and the dissipated power can be formally written as:

(1) $$P=\frac{1}{2}\mu_{0}\chi H^{2}\omega \frac{\omega \tau }{1+{\left(\omega \tau \right)}^{2}}$$

where *µ*_0_ is the magnetic constant, *H* and *ω* are the amplitude of the external magnetic field and its frequency, respectively, *χ* is the magnetic susceptibility of the superparamagnetic particles, and *τ* is the relaxation time of their magnetization. Since *H* and *ω* are limited by unwanted side effects, like arrhythmia, stimulation of muscles, and non-specific heating by eddy currents in the tissue, the magnetic properties of the particles, particularly *χ* and *τ*, have to be tailored to maximize the dissipated power. A large susceptibility in reasonable external magnetic fields can be achieved by choosing superparamagnetic particles with large net magnetic moments. To this end, the particles should also be magnetic single-domain. The relaxation time should fit the inverse frequency of the external magnetic field, *τ* = *ω*^−1^, to obtain maximum available heating. Since it was found experimentally [66] that the particles are quite immobile in tumor tissue, the relaxation time mainly depends on the magnetic anisotropy connecting the favored magnetization directions to the crystal lattice. A review on the use of iron oxide nanoparticles in hyperthermia is given e.g., by Laurent et al. [67].

MRI is based on the nuclear magnetic resonance of protons (hydrogen nuclei) in the tissue. The contrast is given by different relaxation times of the nuclear magnetic moments, depending on the nearest environment usually measured in a sequence of magnetic field pulses. The magnetic stray field of superparamagnetic particles alters the (T_2_ or T_2_*) relaxation time of the protons in the close environment significantly and it can consequently be used as contrast agents. Since a large stray field is caused by particles with a high magnetic moment, again, single-domain superparamagnetic particles are desired. By definition, the large magnetic moment that shall be achieved in reasonably small external magnetic fields is connected to a large susceptibility. In addition, it is also affiliated to other intrinsic and extrinsic parameters, such as magnetic exchange, magnetic order, molecular field, and external applied field [68].

For magnetically targeted drug delivery, a magnetic driving force controls drug distribution and release, which leads to significantly enhanced rates of drug movement and delivery when compared to common approaches, like enhanced permeability and retention (EPR). Because of the biological limitations, like medication’s half-life in the body, a fast and targeted delivery is desired. In addition, side effects can be decreased, since the drugs are only locally concentrated. Magnetically targeted drug delivery is based on an external magnetic gradient field focused on a specific target (e.g., cancerous tissue) to guide magnetic (and improve therapeutic efficiency on tumors.) Again, large magnetic moments/magnetization is desired as the most effective factor for this application, because the magnetic force is proportional to the product of the magnetic field gradient (technically limited) and the magnetic moment of the nanocarrier.

### 2.2. Plasmonic Features

Plasmonic characteristics are associated to the response of nanoparticles to electromagnetic radiation in the specific wavelength ranges of ultra-violet (UV), visible, and near infra-red (NIR) light, which are used for different biomedical applications, including photothermal therapy and imaging approaches [69,70,71].

Photothermal therapy is an active photodynamic therapeutic approach that is based on simulating superficial atoms of plasmonic nanoparticles and localized heating up to a desired temperature. Nanoparticles convert the energy of irradiated light to heat by surface plasmon absorption (SPA). As a result, higher absorption intensity leads to enhanced localized heating and treatment efficiency. When considering the deeper penetration depth of safe NIR as compared to UV and visible light into the body tissues, nanoparticles with surface plasmon absorption at longer wavelengths are desired.

Different bioimaging techniques are powerful non-invasive approaches for accurate early diagnosis of various diseases, like cancerous tumors [72]. For these applications, again, the maximum absorption intensity of nanoparticles leads to higher efficiency. Furthermore, nanoparticles with absorbability in variable ranges of wavelengths shall be simultaneously used in different imaging methods.

**Summary** **1.** *For biomedical applications usually the following magnetic and plasmonic properties are desired*

- *superparamagnetism*
- *large susceptibility/high saturation magnetization (M_S_)*
- *suitable relaxation time*
- *Maximum surface plasmon absorption (SPA) intensity*
- *Plasmon resonance absorption peak (PRA) in the near infra-red (NIR) regime*

Both superparamagnetism and relaxation time in tumor tissue can be tailored by the magnetic anisotropy. The relaxation time in media with low viscosity mainly depends on the size (which influences the occurrence of superparamagnetism as well) and surface design. Both SPA intensity and peak position directly depend on the chemical composition, crystal structure, surface properties, morphology, and size of nanoparticles.

## 3. Structure Design

Magnetic-plasmonic heterodimer nanoparticles were designed in various structures, depending on their desired use. The appropriate structure of particles was identified by obtaining maximum functionality in intended applications. Different factors including particles size and shape, components phases, chemical composition, crystal structure, etc. were considered and variated as effective parameters of heterodimers structure design. Among them, composition nature (metallic vs. nonmetallic) and morphology of components, as well as their junction interfaces, are the most critical factors. Accordingly, the following discussion of structural design of recently reported heterodimer structures is systematically reviewed in two parts: electronic nature of components and interfacial morphology, respectively.

### 3.1. Electronic Nature of Components

Both magnetic and plasmonic components could be comprised of metallic or nonmetallic natures. Therefore, we classify the heterodimer structures according to the three possible combinations of different electronic natures, which are presented below: metallic-metallic, metallic-nonmetallic, and nonmetallic-nonmetallic.

#### 3.1.1. Metallic—Metallic

Noble metals nanoparticles consisting of Au, Ag, Pt, Pd, Cu, and Ti were frequently used as plasmonic nanomaterials in the synthesis of heterodimer structures [73,74]. Although most of them, particularly Au and Ag nanoparticles, demonstrate insignificant magnetic features, they also led to a functionality enhancement of heterodimers in biomedical applications when combined with a ferromagnetic material. Metallic nanoparticles of the ferromagnetic elements Fe, Co, and Ni nanoparticles had been introduced as efficient candidates for the magnetic part of heterodimers because of their supreme magnetic characteristics, like reasonable saturation magnetization (M_S_) and anisotropy [75]. However, the utilization of metallic magnetic components was commonly limited by their insufficient chemical stability, so that there was a need for well-designed synthesis processes and the use of stabilizing surfactants, such as oleic acid, polyethylene glycol (PEG), and poly(N-vinylpyrolidone) (PVP) [76,77]. Furthermore, the biocompatibility of these particles, especially Co and Ni, for biomedical applications was inadequate and additional improving treatments, like surface coating with silica, biomolecules, and polymers are necessary [78,79].

**Figure 1:**
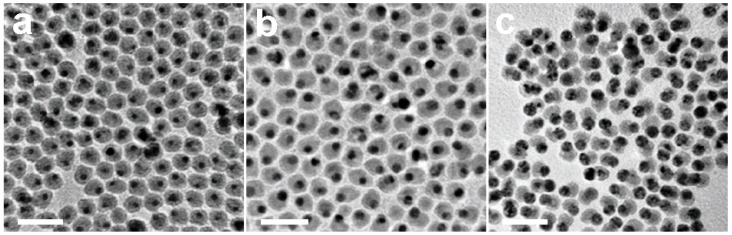
Transmission electron microscopy (TEM) images of Fe-Au heterodimers with different particles sizes. 15–4 nm Fe-Au heterodimers (**a**), 7–13 nm Fe-Au heterodimers (**b**), and 11–10 nm Fe-Au heterodimers (**c**), scale bars: 50 nm. Reproduced with permission from [81], copyright (2016) Royal Society of Chemistry.

The reported work in 2007 by Wetz et al. on the synthesis of Co-Au heterodimer nanorods based on the controlled nucleation and growth of Au nanoparticles at the surfaces of Co nanorods was an important step forward to introduce a new generation of hybrid materials [80]. Recently, Jiang et al. synthesized Fe-Au heterodimers through ultrasmall Fe (bcc) and Au seeds with the possibility of controllable transformation to a biocompatible Fe_3_O_4_-Au structure [81]. The size and chemical stability of these nanoparticles were adjusted using oleic acid as chelating agent, so that the diameters of Au and Fe particles were in the range of 4–10 and 11–15 nm, respectively (Figure 1). Moreover, multifaceted Fe-Pd heterodimers are simply assembled based on a sequential reduction process of Fe and Pd metallic nanoparticles [82]. In this work, the diameter of the Fe particles was in the range of 7–15 nm and the mean diameter of Pd nanoparticles was around 1 nm. In addition to biomedical applications, metallic-metallic heterodimers, such as Ni-Au nanostructures, could be utilized in bio- and photo-catalysis as well, owing to their multifunctional properties [83].

In comparison with single-element magnetic particles, magnetic bimetallic compounds offered the possibility to tailor the magnetic characteristics, such as M_S_, anisotropy, coercivity (H_C_), power square, and remanence-to-saturation ratio (M_r_/M_S_). In addition, they exhibited enhanced chemical stability [84], making bimetallic magnetic nanoparticles the more appropriate candidates for magnetic components of heterodimers. Bimetallic magnetic structures with compositions of M_x_P_y_ (M = Fe, Co, Ni, and P = Pt, Pd) have been growingly utilized in bioapplications, such as MRI contrast agents [85], cancer therapy treatment killing Hela cells [86], and the prevention of the growth and spread of tumors [87,88]. As a same trend, heterodimer structures composed of magnetic bimetallic and noble metals components presented excellent and even occasionally unforeseen features. For instance, despite the presence of diamagnetic Au nanoparticles in CoPt_3_-Au heterodimers leading to a decrease of the net magnetic properties, CoPt-Au heterodimer nanostructures showed enhanced magnetization [86]. Moreover, simultaneously strengthened magnetic and plasmonic features of FePt-Au and FePt-Ag heterodimers were reported [89,90,91]. Recently, Lopez-Ortega et al. developed metallic Ag@FeCo hybrid nanostructures by combining hot injection and polyol approaches in high boiling point solvents [92] in chemical synthesis. They presented promising results in enhanced plasmonic and magneto-optical properties, which were comparable to the best reported results for nanohybrids that were fabricated using physical methods, like lithography. The magnetic control of light polarization was significantly increased, which is very useful for optical communication, sensing, and imaging applications. Furthermore, the reported achievements confirmed the capability of chemical synthesis methods to control size, shape, and compositions of nanohybrids [92].

#### 3.1.2. Metallic—Nonmetallic

Metallic–nonmetallic structures form the main category of magnetic-plasmonic heterodimers because of their optimum characteristics, in particular magnetic properties and chemical/biological stability. Nonmetallic components were often considered as a host component due to their higher hydrophilicity when compared to metallic particles, leading to a higher stability of the combined structure.

According to the literature, most of the metallic-nonmetallic heterodimers had been designed based on various well-defined nonmetallic magnetic transition metal oxides, particularly ferrites. Several works on using magnetic ferrites, such as Fe_3_O_4_, CoFe_2_O_4_, MnFe_2_O_4_, and NiFe_2_O_4_ combined with nanoparticles of noble metals, like Au, Ag, Pt, and Pd had been reported [93,94]. Nowadays, magnetite (Fe_3_O_4_) nanoparticles play a key role in biomedical applications as they are approved by the United States (US) Food and Drug Administration (FDA) for distinct medical treatments and currently investigated in clinical trials [95]. In the European Union, functionalized magnetite nanoparticles are approved for the hyperthermia treatment of brain tumors. Following this route, different heterodimer structures on the basis of magnetite particles, such as Fe_3_O_4_-Au, Fe_3_O_4_-Ag, Fe_3_O_4_-Pt, and Fe_3_O_4_-Pd with various particles sizes and morphologies were designed for bioapplications [96,97,98,99,100,101,102,103]. Among them, Fe_3_O_4_-Au heterodimers attracted the most attention for catalytic, analytical, and biomedical applications [104].

**Figure 2:**
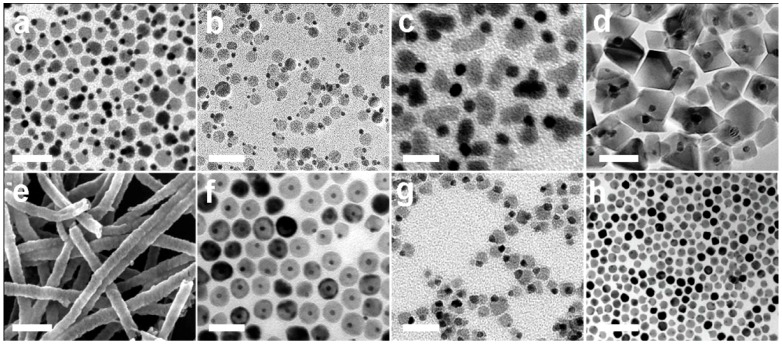
Microscopic images of different metallic-nonmetallic heterodimers. TEM image of Fe_3_O_4_-Au heterodimers, scale bar: 40 nm, reproduced with permission from [110], copyright (2015) American Chemical Society (**a**), TEM image of Fe_3_O_4_-Ag heterodimers, scale bar: 40 nm, with permission from [111], copyright (2017) Elsevier (**b**), TEM image of Fe_3_O_4_-Pt heterodimers, scale bar: 30 nm, with permission from [112], copyright (2013) American Chemical Society (**c**), TEM image of Fe_3_O_4_-Pd heterodimers, scale bar: 30 nm, with permission from [113], copyright (2011) Royal Society of Chemistry (**d**), SEM image of CoFe_2_O_4_-Pd heterodimers, scale bar: 300 nm, with permission from [117], copyright (2016) Royal Society of Chemistry (**e**), TEM image of MnO-Au heterodimers, scale bar: 40 nm, with permission from [121], copyright (2014) American Chemical Society (**f**), TEM image of FePt-CdS heterodimers, scale bar: 40 nm, with permission from [123], copyright (2009) American Chemical Society (**g**), and TEM image of CdO-FePt heterodimers, scale bar: 100 nm, with permission from [73], copyright (2014) American Chemical Society (**h**).

For instance, Fantechi et al. synthesized Fe_3_O_4_-Au heterodimers through Au seed mediated thermal decomposition and precisely controlled the size, morphology, Fe/Au ratio, and crystallinity by deliberately changing of iron initial resources (Figure 2a) [105]. In other works, a considerable alteration of magnetic and plasmonic features of Au-iron oxide heterodimers was reported as a consequence of size and shape controlling of Au nanoparticles [106,107]. In spherical Fe_3_O_4_-Au heterodimer structures that was designed by Landgraf et al. not only an improvement in biocompatibility and a reduction of side effects of magnetic nanoparticles were observed, but also their performance in MRI applications was optimized from the path of interactions with proteins in biologic fluids [108]. Additionally, based on the magnetic properties of Fe_3_O_4_ and the plasmonic features of Au nanoparticles, Fe_3_O_4_-Au heterodimers could act as ideal contrast agents for multimodal bioimaging techniques, including CT, MRI, photoacoustic (PA), optical microscopy (OM), transmission electron microscopy (TEM), and surface-enhanced Raman spectroscopy (SERS) [109]. Read et al. smartly used Fe_3_O_4_-Au heterodimers as host seed platform to assemble the stable structure of Fe_3_O_4_-Au-Ge heterodimers, while a Fe_3_O_4_-Ge structure showed insignificant physicochemical stability (Figure 2a) [110].

Recently, Ding et al. synthesized Fe_3_O_4_-Ag heterodimers for magnetic hyperthermia applications with remarkably enhanced efficiency of magnetic heating when compared to individual Fe_3_O_4_ nanoparticles, which could be attributed to the much larger heat transfer efficiency of metallic Ag attachments as compared to nonmetallic magnetic nanoparticles (Figure 2b) [111]. In addition, the performance of hybrid nanostructures in photothermal therapy was improved in comparison with both individual Fe_3_O_4_ and Ag nanoparticles and they exhibited better biocompatibility.

In recent years, several heterodimer nanostructures that were composed of Fe_3_O_4_ and metallic Pt and Pd nanoparticles were synthesized (Figure 2c,d) [112,113]. Although paramagnetic nanoparticles of Pt and Pd were nominated as plasmonic agents, owing to their more proper biocompatibility and stability, rather than diamagnetic Au and Ag particles, they were only limitedly utilized because of the rather complicated synthesis and attaching process. Furthermore, Fe_3_O_4_-Cu heterodimers in submicron size range exhibited pseudo-superparamagnetic behavior [114].

Other ferrite structures could be actively selected as a magnetic component. For instance, MnFe_2_O_4_-Ag heterodimers were seed-mediated fabricated via elaborate control of synthesis parameters, such as reaction temperature, pH, and time [94]. Moreover, CoFe_2_O_4_-Au, CoFe_2_O_4_-Ag, and CoFe_2_O_4_-Pd heterodimers had been synthesized for antibacterial and photocatalysis applications (Figure 2e) [115,116,117]. The latter system shall be presented here in some more detail. CoFe_2_O_4_-Pd heterodimer nanostructures were successfully synthesized with different particles sizes (from 7 to 40 nm) using pre-synthesized magnetic nanoparticles of cobalt ferrite (CoFe_2_O_4_) [118,119] as the host seeds to reduce ultra-small Pd particles. CoFe_2_O_4_ nanoparticles were randomly decorated with metallic Pd components with a mean diameter of 2 nm [120]. Size and weight percent of Pd nanoparticles of heterodimers were controlled through adjusting synthesis parameters, such as pH, reaction temperature, and initial concentration of palladium resources. We found that both magnetic and plasmonic characteristics of CoFe_2_O_4_-Pd heterodimers (Pd amount was varied in the range of 1.5–3%wt) were significantly improved when compared to individual CoFe_2_O_4_ nanoparticles, which are presented in next sections.

There were only a few reports on applying other metal-oxide structures for a magnetic building block. Biocompatible SiO_2_-covered MnO-Au heterodimers were synthesized by Schick et al. for use in bioapplications (Figure 2f) [121]. Although superparamagnetic MnO nanoparticles indicated non-highlighted magnetization, they brought considerable benefit to contrast the enhancement in MRI and particularly fluorescence and confocal laser scanning imaging [121,122]. On the other hand, the combination of metallic magnetic and nonmetallic plasmonic parts was occasionally reported. For example, FePt-CdS hybrid structures were assembled by using magnetic FePt bimetallic nanoparticles and plasmonic CdS quantum dots (Figure 2g) [123,124]. Moreover, the synthesis of FePt-CdO heterodimers was introduced for plasmonic applications (Figure 2h) [73].

#### 3.1.3. Nonmetallic—Nonmetallic

In order to synthesize heterodimer structures, nonmetallic magnetic particles were frequently utilized, but so far only a few plasmonic components with nonmetallic nature were employed. The main class of nonmetallic plasmonic nanomaterials is quantum dots (QDs) with numerous functionalities for biomedical applications, such as drug delivery, especially for small molecules, like 5-FU [125,126], biosensing [127,128,129,130], and multimodal imaging [131,132]. Since quantum dots normally show insufficient biocompatibility and colloidal stability, their combination with nonmetallic magnetic nanoparticles could be helpful to overcome drawbacks. Lee et al. synthesized Fe_3_O_4_-QD heterodimers that are based on a one pot self-assembly method with simultaneous improvements in Hela cells uptake and labeling for in-vitro/in-vivo imaging [133].

**Figure 3:**
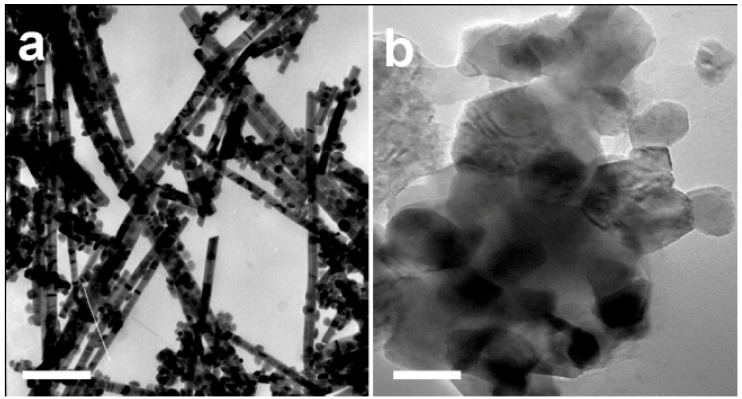
TEM images of different nonmetallic-nonmetallic heterodimers. Fe_2_O_3_-CdS heterodimers, scale bar: 300 nm, reproduced with permission from [134], copyright (2009) American Chemical Society (**a**), and CoFe_2_O_4_-ZnO heterodimers, scale bar: 50 nm, with permission from [137], copyright (2013), Elsevier (**b**).

Investigation of Fe_2_O_3_-CdS heterodimers confirmed the synergistic emergence of magnetic and photoluminescence properties in a unique structure (Figure 3a) [134]. In addition to common quantum dots, some other nonmetallic structures, such as Ti oxides and Zn oxides, consequently showed plasmonic features and were used as a plasmonic component of heterodimers [135]. Despite the successful synthesis of Fe_3_O_4_-ZnO [136] and CoFe_2_O_4_-ZnO heterodimers (Figure 3b) [137], an investigation of their biological features is missing as of yet. Recently, some new generations of plasmonic materials, like graphene QD, were employed in the Fe_3_O_4_-graphene QD heterodimers for bioapplications [84,138].

Although the most efficient magnetic-plasmonic nanoparticles are composed of two or more phases as a combination of magnetic and plasmonic phases, recently some interesting works were presented based on single-phase magnetic-plasmonic nanomaterials. Nickel-based phases, like metallic Ni nanoparticles and NiO nanostructures, were the main candidates for single-phase multifunctional nanoparticles for biomedical applications, because they indicate significant ferromagnetic and plasmonic features [139,140]. Furthermore, there were some reports on employing alloyed structures as single-phase magnetic-plasmonic nanoparticles. For instance, Messina et al. tuned the composition of Ni_x_Au_y_ alloy nanoparticles to perform mixing of both magnetic and plasmonic properties [141]. However, the magnetization of this single-phase (for example Ni_67_Au_33_) was remarkably decreased when compared to pure Ni nanoparticles. Amendola et al. fabricated iron-doped silver nanotruffles with simultaneous magnetic and plasmonic characteristics [142]. Investigation of their electronic structure confirmed the single-phase formation of FeAg with significant magnetic and plasmonic properties for photothermal heating applications. The future developments of single-phase magnetic-plasmonic structures can provide promising advances for biomedical applications.

**Summary** **2.** *Metallic-metallic heterodimers can exhibit improved magnetic and plasmonic characteristics outperforming nonmetallic nanoparticles, while practical restrictions in their chemical and biological stability limit their use in biomedical applications. Various structures of metallic-nonmetallic heterodimers were increasingly employed in and developed for bioapplications owing to a variety of tailored features from magnetization to physiochemical and biological stability. Despite attractive characteristics of nonmetallic-nonmetallic heterodimers, so far only a few research groups focused on them. In fact, a bright future in bioapplications could be expected after more studies on this novel kind of magnetic-plasmonic heterodimers.*

### 3.2. Interfacial Morphology

**Figure 4:**
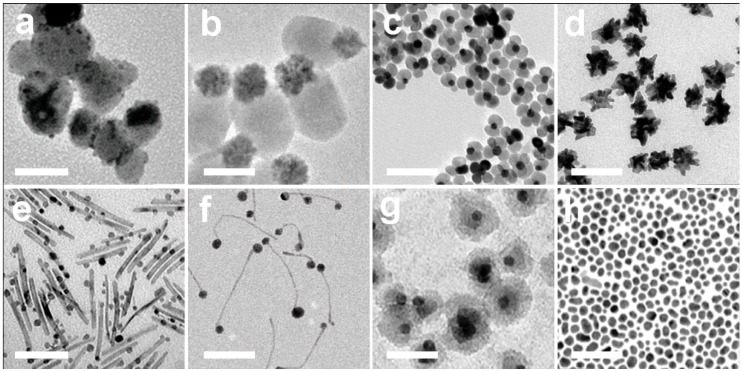
TEM images of heterodimers with different interfacial morphologies. Random-decorated Fe_2_O_3_-Pd heterodimers, scale bar: 30 nm, reproduced with permission from [32], copyright (2015), Royal Society of Chemistry (**a**), Janus Fe_3_O_4_-antibody dye heterodimers, scale bar: 100 nm, with permission from [143], copyright (2018) Elsevier (**b**), Flower-shaped MnO-Au heterodimers, scale bar: 50 nm, with permission from [121], copyright (2014) American Chemical Society (**c**), Star-shaped FeO_x_-Au heterodimers, scale bar: 100 nm [107], published by The Royal Society of Chemistry (**d**), Rod-shaped Fe_2_O_3_-TiO_2_ heterodimers, scale bar: 200 nm, with permission from [144], copyright (2009) Royal Society of Chemistry (**e**), Dumbbell-shaped FePt-Au heterodimers, scale bar: 30 nm, with permission from [90], copyright (2011) Spiringer Nature (**f**), Mosaic Fe_3_O_4_-Au(thiol)@SiO_2_ heterodimers, scale bar: 50 nm, with permission from [108], copyright (2015) Elsevier (**g**), and Core-shell Fe_3_O_4_@Au heterodimers, scale bar: 50 nm, with permission from [145], copyright (2005) American Chemical Society (**h**).

Junction mode and interfacial morphology of heterodimer structures are the most effective factors for their design, characteristics, and final performance. Interfacial morphology depends on the composition, individual shape, and surface properties of components. Hitherto, several magnetic-plasmonic heterodimers with different interfacial morphologies were synthesized. The most desired morphologies included random-decorated, dumbbell-like shape, mosaic, rod-shape, and Janus beads (Figure 4) [143,144,145]. Per definition, core-shell structures could be categorized as heterodimers as well, but due to their great diversity they were classically treated as an individual group and they are not part of this review. Furthermore, there are some reports on the heterodimers with specific interfacial morphologies, such as flower and star-shape. In the following, recent works on each mentioned category are briefly reviewed.

#### 3.2.1. Randomly Decorated

Seed mediated synthesis methods of heterodimers mostly results in the random joining of components so that the larger building block is typically considered as a host of arbitrary deposition of guest particles. In such junction morphology, components could be used with different particles shape, such as spherical [111], cubic [89], and irregular non-geometric [146,147,148]. Ding et al. shape-controlled fabricated Fe_3_O_4_-Ag heterodimers in two formats: randomly decorated Fe_3_O_4_ with spherical Ag nanoparticles and core-shell structures through deliberate changing the concentration of the Ag precursor [111]. Hodges et al. reported the synthesis of Fe_3_O_4_-Ag heterodimers of randomly decorated and core-shell morphologies. Fe_3_O_4_ and Ag particles size and shapes were entirely controlled in linearly variations at the ranges of 7–24 nm and 3–11 nm, respectively [149].

It was found that different morphologies led to differences in magnetic and biocompatibility properties with the randomly decorated structures demonstrating enhanced magnetization, biocompatibility, and specific absorption rate (SAR) in hyperthermia applications [111]. Additionally, different plasmonic features arise from the morphologies changes, so that the localized surface plasmon resonance (LSPR) peak of random decorated heterodimers shows a blue shift to shorter wavelengths, while the LSPR peak of core-shell structures is in agreement with that of individual metallic Ag nanoparticles [150].

#### 3.2.2. Dumbbell-Like Shape

Fe_3_O_4_-Au heterodimers that were synthesized by Yu et al. in 2005 were introduced as a first example of dumbbell-like structures [151]. In most dumbbell-like heterodimers, magnetic uniaxial nanorods acted as host for spherical metallic nanoparticles as plasmonic guests that are symmetrically attached to the host surfaces [97,100,152]. Dumbbell-like heterodimers demonstrated desirable performances in photo- and bio-catalysis applications due to their higher symmetry and larger free surface areas when compared to randomly decorated structures [106]. Hou’s research group synthesized FePt-Au heterodimers with random decoration and dumbbell-like morphologies for biomedical and catalysis applications [89,90] based on seed mediated technique of non-spherical magnetic particles [153]. In both cases, the plasmonic properties were affected by the interfacial morphology and their UV-Vis absorption peaks were slightly red-shifted in comparison with Au nanoparticles [154,155]. In fact, UV-Vis spectroscopy of individual Au nanoparticles shows a well-pronounced peak at 520 nm, while for randomly decorated and dumbbell-like heterodimers, it is shifted to 525 and 528 nm, respectively, owing to electron transfer from the Au surface to the FePt component [90].

#### 3.2.3. Mosaic

**Figure 5:**
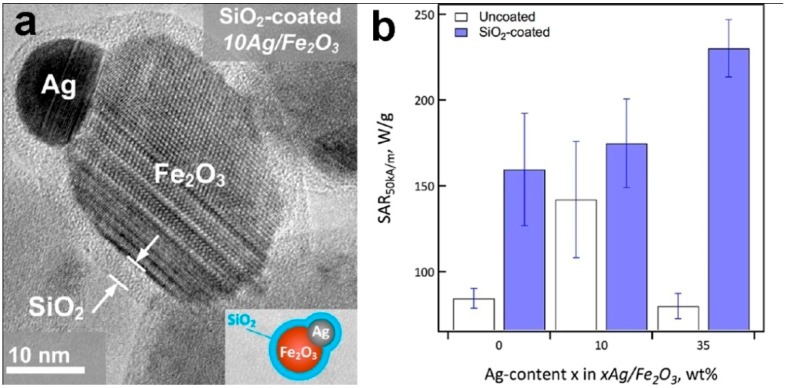
SiO_2_ coated Fe_2_O_3_-Ag heterodimers. HRTEM image of heterodimer structure, with 10 wt% Ag (**a**) and specific absorption rate (SAR) values of uncoated and SiO_2_ coated heterodimers with different Ag concentrations (**b**). Reproduced with permission from [17], copyright (2013) American Chemical Society.

Mosaic structures are normally utilized when heterodimers need to be covered by a shell or put in a matrix. The main reasons for these designs are improvement of biocompatibility, physicochemical stability, and advanced functionality. In several reported mosaic heterodimers, silica is considered as matrix structure, such as FePt-Au@SiO_2_ and FePt/Fe_3_O_4_-CdSe@SiO_2_ nanohybrids [14,156]. Sotiriou et al. fabricated Fe_2_O_3_-Ag heterodimers on SiO_2_ mosaic shell to use in magnetic hyperthermia (Figure 5a) [17]. The SAR was appreciably increased for the silica coated structures for all Ag concentrations (Figure 5b). In addition, an unwanted ferromagnetically blocked fraction was decreased by the silica shell due to the reduced interactions between the magnetic parts. In Au(thiol)-Fe_3_O_4_@SiO_2_/PEG heterodimers that were synthesized by Landgraf et al., silica/PEG possessed not only enhanced biocompatibility and chemical stability, but also improved their efficiency as MRI contrast agents [108]. Moreover, the SiO_2_ matrix of designed MnO-Au@SiO_2_ heterodimers for bioimaging led to advances in MRI contrast and fluorescence microscopic cells tracking [121]. Recently, Fe_3_O_4_-graphene QD heterodimers with silica mosaic cover were developed with outstanding magnetic, plasmonic, and biocompatibility properties to utilize in drug delivery, hyperthermia, and photothermal therapy applications [138].

Further mosaic coatings were employed to support heterodimers. For instance, Giang et al. synthesized Fe-Au hybrids in a porous Fe_3_O_4_ mosaic coating with tunable magnetization and surface plasmon absorption [81]. Additionally, a wide range of biomolecules and polymers could be performed as a matrix of magnetic-plasmonic structures, which were frequently investigated in reviews of polymeric coatings [157,158]. These structures are commonly used as nanocarriers for drug delivery [95,159].

#### 3.2.4. Rod-Shape

Specific characteristics of rod-shape structures are a possible reason to outperform spherical particles. For instance, the shape anisotropy remarkably affected hyperthermia efficiency [160,161,162]. In 2007, Wetz et al. pioneered rod-shape heterodimers by synthesizing Co-Au structures via nucleation control of Au upon Co seeds [80]. Yang et al. used the electrospinning-presynthesized CoFe_2_O_4_ nanotubes as host seeds to fabricate rod-shape CoFe_2_O_4_-Pd heterodimers for H_2_O_2_ detection [117].

The synthesis of rod-shape Fe_3_O_4_-Au heterodimers for bioapplications was reported in several works [101,163,164,165,166]. Fe_3_O_4_-Pt heterodimers of rod-shape as well as core-shell morphologies were fabricated using Pt seeds and they exhibited significantly enhanced plasmonic properties for photothermal therapy when compared to their single components [167]. In the same direction, the variation of magnetic and particularly plasmonic features as a function of morphologies was reported for rod-shape NiPt-CdS structures [168]. However, although the rod-shape heterodimers brought enormous advantages, they were faced with limitations for in vivo applications, like drug delivery, because of their problematic targeting and transfer in vessels [95].

#### 3.2.5. Janus Beads

Janus beads are particles consisting of two or more different natures in a unique structure. Although this definition could be associated to any heterodimers, Janus bead usually refers to combined particles with different surface characteristics and very similar dimensions and shape. Nanoparticles with different surface coatings [169], adsorption [170], electrochemical activity [171], and electrical charge [172], as well as amphiphilic surfaces [173] were introduced as the most common categories of Janus beads. Various organic (biomolecules and polymers) and inorganic (oxides and ceramics) materials could be utilized to form Janus beads [174,175]. Self-assembly of Fe_3_O_4_-Au Janus beads improved magnetic, plasmonic, and biocompatibility properties and presented two different approaches of active and passive drug delivery [108,176]. Jishkariani et al. reported the fabrication of Janus hybrids with two opposite surface properties, hydrophobic and hydrophilic, by attaching Au and Pt nanoparticles on Fe_3_O_4_ surfaces using intermediary interactions of polar-/nonpolar-dendrons [176]. These beads could be used to carry many types of bioagents, like drugs and antibodies.

Reguera et al. synthesized Fe_3_O_4_-Au Janus heterodimers of star-like morphology [109]. The Fe_3_O_4_ nanoparticles were jointed to star-shaped Au seeds without morphology change, so that the plasmonic properties of Janus beads were noticeably enhanced when compared to randomly decorated structures. Recently, Chang et al. designed Janus beads with magnetic-fluorescence sensitivity, while sensitivity is increased via antibody attaching to the carboxyl-functionalized Fe_3_O_4_-dye heterodimers for rapid identification of foodborne bacteria [143].

#### 3.2.6. Further Specific Shapes

**Figure 6:**
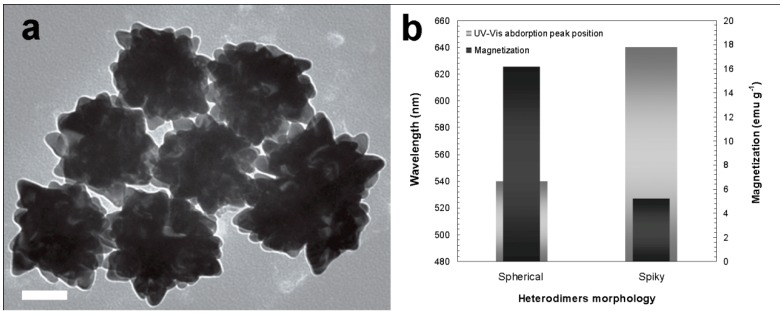
Fe_3_O_4_-Au heterodimers. TEM image of spiky-shaped structure, scale bar: 100 nm (**a**) and Variation charts of magnetization and UV-Vis absorption peak position as a function of morphology (**b**). Adapted from reference [177].

Although morphology is one of the most important parameters to determine the final features and performance of heterodimers, most reported works were carried out on common shapes, in particular, spherical. In recent years, there were a few works on the identification of optimum synthesis conditions for designing heterodimers with specific morphologies, including star- and flower-like shapes [107]. Zhou et al. synthesized star-like Fe_3_O_4_-Au heterodimers by the use of Fe_3_O_4_ seeds and deposited star-shaped Au nanoparticles through the hydroquinone approach (Figure 6a) [177]. Magnetic-plasmonic investigations revealed their improved plasmonic characteristics in comparison with spherical particles as a significant red-shift in UV-Vis absorption. However, magnetization of star-shaped heterodimers dramatically dropped (Figure 6b). Moreover, in the case of Fe_3_O_4_-Ag heterodimers, flower-shaped structures showed the largest magnetic anisotropy and highest blocking temperature (T_B_) among various morphologies [178]. Fantechi et al. elaborately designed flower-like Fe_3_O_4_-Au heterodimers, which led to substantial changes of magnetic and plasmonic behaviors [105]. These structures were assembled using Au seeds and by controlling the [Fe]/[Au] molar ratio, heating rate, reaction time, and surfactant kinds. In addition, the successful synthesis of flower-shaped MnO-Au nanohybrids was recently reported [121]. In addition, the synthesis of designed heterodimers with other specific shapes, like nanosandwich, nanodisk, and nanodome were reported. Gonzales-Diaz et al. synthesized magnetic-plasmonic Au/Co/Au nanosandwiches consisting of sputtered trilayer films using the colloidal lithography (CL) method [179]. The heterodimers simultaneously exhibited strongly localized surface plasmon resonance, magnetic, and magneto-optical properties. In this case, the plasmonic nanostructures could be directly controlled by an external magnetic field. Moreover, Au/Co/Au, Au/SiO_2_/Co/Au, and Au/Co/SiO_2_/Au hybrid structures with nanodisk morphology were fabricated by Banthi et al. using CL and evaporation methods [180]. Electromagnetic field of magneto-optical components (Co) and non-magneto-optical parts (Au) has been controlled by adjusting the position of the SiO_2_ dielectric layer in the hybrid structures, which lead to providing a system with enhanced magneto-optical activity and moderate absorption.

Recently, new types of magnetic-plasmonic heterodimer nanostructures, namely Co/Au and Co/Fe nanodomes, were proposed by Nogues’s research group [181]. Fe (or Co)-Au nanodomes demonstrated larger photothermal conversion efficiency when compared to pure Au nanodomes and other reported shapes, like Au nanorods and nanoshells as a result of strong optical absorption, minimized scattering, and low optical anisotropy. The Fe and Au semishells, respectively, provided a very intense T_2_ contrast in nuclear magnetic resonance (up to 15-fold larger than commercial iron oxide contrast agents), and active fluorescent and X-ray contrast for magnetically guided and amplified photothermal therapies [181]. In addition, this research group presented interesting thermometry nanotechnology based on Fe (or Co)-Au nanodomes [182]. The temperature variations during magnetic and optical hyperthermia treatments were detected by viscosity variations around the nanodomes based on the identification of phase lag between the optical signal and driving magnetic field. The resolution of temperature detection was around 0.05 °C similar to state-of-the art luminescent nanothermometers, which were highlighted as a low cost temperature detection system for biomedical applications [182].

**Summary** **3.** *Various morphologies and interface structures in heterodimers can be designed today. For randomly decorated heterodimers, the synthesis process is usually simpler than that of other morphologies and their magnetic and plasmonic properties can be more appropriate. Therefore, randomly decorated heterodimers could be the most enthralling selection for biomedical applications, particularly in vivo treatments, owing to their significant functionality and biocompatibility.*

Heterodimer structures with non-geometric specific shapes may further improve their performance. However, this research is still in the beginning, so most of the reported work focused on identification of fundamental factors in morphology control, which is essential to start with practical studies in the future.

## 4. Tailored Properties of Selected Heterodimers

Nowadays, the main motivation of design and synthesis magnetic-plasmonic heterodimer nanostructures is the improvement in their efficiency for various applications. Nanoparticles features could be defined as an interface point between the design and final performance of structures. In the following, the most effective features and how they can be tailored in magnetic-plasmonic heterodimers are concisely reviewed.

### 4.1. Magnetic Features

Many applications of magnetic-plasmonic heterodimers are attributed to their magnetic properties. As stated in Section 2, magnetic applications depend on some key parameters, such as high susceptibility or saturation magnetization (M_S_), superparamagnetism connected to vanishing coercive field (H_C_) and remanent magnetization (M_r_), and suitable anisotropy. Since most of the plasmonic materials exhibit small magnetic moments due to their paramagnetic or diamagnetic nature, the net magnetization of heterodimers mostly decreased when compared to the individual magnetic components. Furthermore, magnetic dipole interactions between magnetic nanoparticles are attenuated in heterodimers, leading to changes of H_C_, M_r_, and M_S_. In addition, surface effects, including both structural changes and electronic hybridization effects, can significantly modify the magnetic moments of the magnetic component and the anisotropy [183]. For instance, comparing FePt-Au heterodimers with FePt particles reveals that blocking temperature of heterodimers (25 K) is reduced from 40 K to 25 K as a result of anisotropy decrease and surface texture changing [90,184]. However, it has been shown that, while depending on the surface termination, an increasing anisotropy of this system is possible [183].

The particles size of plasmonic component could affect the magnetization of structures as well. Jiang et al. reported that the saturation magnetization of Fe-Au heterodimers increased up to 6 and 12% as a function of size variations of Au nanoparticles from 4 to 7 and 10 nm, respectively because of the different surface structures [81,185]. In addition, the shape of magnetic field dependent magnetization curves was shown to depend on Au particles, so that the saturation field (H_S_) value of decorated structures was extremely enhanced due to surface spin canting. In the case of Fe-Pd heterodimers, a decrease of M_S_ was reported when compared to individual Fe particles [82]. Heterodimers that were composed of nonmetallic plasmonic components, like QDs, revealed more devastating impacts on magnetic properties. For instance, in FePt-CdS [123] and CoFe_2_O_4_-ZnO [137] structures, the magnetization is severely diminished when compared to the one of the bare magnetic constituents.

Ding et al. showed a negligibly reduced magnetization of Fe_3_O_4_-Ag hybrids as compared to Fe_3_O_4_ nanoparticles [111]. Furthermore, Jiang et al. reported that the coercivity of Fe_3_O_4_-Ag heterodimers was identical to that of individual Fe_3_O_4_ particles, while hybrids magnetization was slightly decreased [186]. On the other hand, the presence of Ag nanoparticles in MnFe_2_O_4_-Ag structures led to a strong reduction of magnetization [94]. Moreover, the magnetization lowering of Fe_3_O_4_-Au heterodimers was reported in many works [105,109,143].

**Figure 7:**
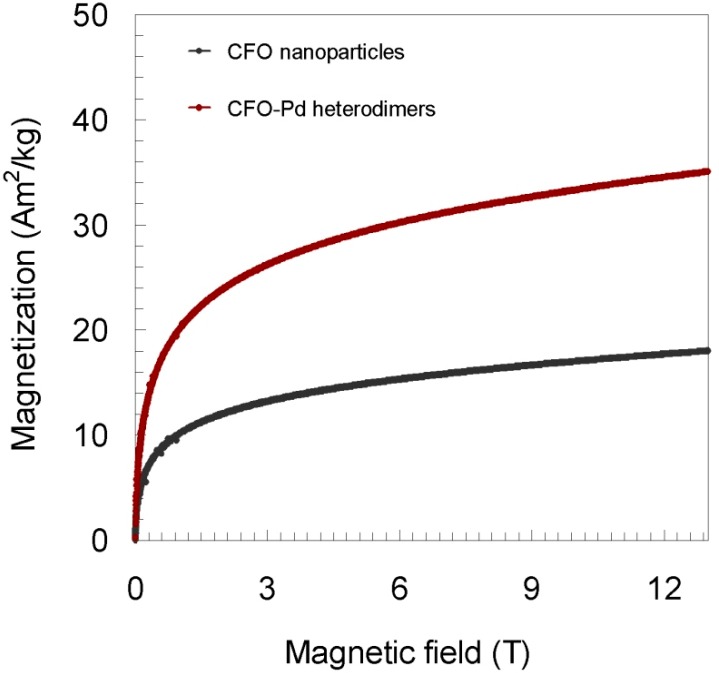
M-H curves of superparamagnetic CoFe_2_O_4_ nanoparticles (CFO, mean hydrodynamic size: 24 nm) and CoFe_2_O_4_-Pd (CFO-Pd, mean particles size of Pd: 2 nm) heterodimers measured by vibrating sample magnetometer (VSM) magnetometry at 300 K.

**Figure 8:**
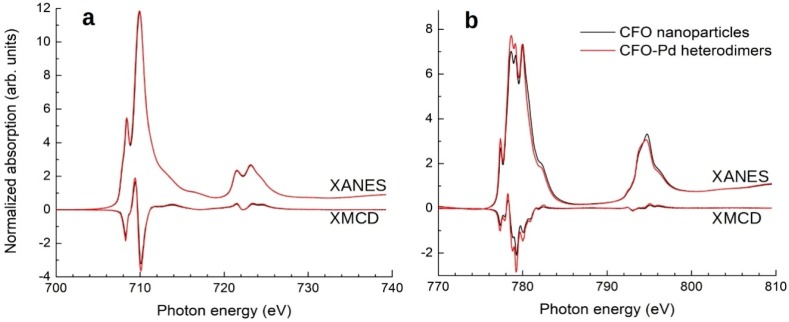
X-ray absorption near-edge structure (XANES) and magnetic circular dichroism (XMCD) of bare CoFe_2_O_4_ nanoparticles (CFO, black lines) and after Pd decoration (CFO-Pd, red lines). Measurements were performed at 5 K in a magnetic field of 6 T at the Fe L_3,2_ absorption edges (**a**) and Co L_3,2_ absorption edges (**b**).

In order to investigate the changes in the magnetic properties in more detail, in addition to common averaging magnetometry methods using vibrating sample magnetometer (VSM) or superconducting quantum interference device (SQUID), the element-specific magnetization was studied through advanced techniques, like X-ray magnetic circular dichroism (XMCD). For example, according to calculated parameters based on magneto-optic sum rules [187,188,189], such as spin, orbital, and total magnetic moments (m_s_, m_l_, and m_tot_), Fe_3_O_4_-Ag heterodimers have lower values than Fe_3_O_4_ nanoparticles, owing to spin injection from Fe_3_O_4_ to Au particles and associated spin contribution [190]. Frequently, diamagnetic plasmonic compounds, like metallic Au, Ag, and Cu particles and QDs, showed a weakening of the magnetization of the ferromagnetic/ferrimagnetic parts. However, some paramagnetic noble metals, like Pd and Pt particles, could prevent the decrease of magnetic properties and/or even improve them occasionally. Synthesized Fe_3_O_4_-Pd heterodimers by Hyon’s research group presented significant magnetization that was identical to bare Fe_3_O_4_ nanoparticles [113]. Moreover, based on VSM and XMCD results of randomly decorated CoFe_2_O_4_-Pd heterodimers, it was found that their magnetization was considerably increased in comparison with individual CoFe_2_O_4_ nanoparticles (Figure 7) [120]. By element-specific analyses of the magnetic response of XMCD it could be deduced that the Co^2+^ ions in CoFe_2_O_4_ play a crucial role in the magnetization enhancement (Figure 8). Interaction of paramagnetic Pd atoms and magnetic cations of Fe and Co at the interface of CoFe_2_O_4_/Pd nanoparticles could be introduced as a reason of this increase, which led to improved heterodimers’ efficiency in related applications. In addition, calculated magnetic anisotropy of CoFe_2_O_4_ nanoparticles with different sizes was enhanced up to three times as a result of Pd decoration. This achievement can be considered as an important step-forward to design the new generations of heterodimers for magnetic applications.

### 4.2. Plasmonic Properties

The main aim of heterodimers synthesis is the combination of both plasmonic and magnetic properties in a single structure. Although noble metals, like Au and Ag, are known as ideal candidates for plasmonic applications, in the bulk state they show negative effects on magnetization while their nanometric particles have a higher magnetic susceptibility [191,192] and more suitable conditions to combine with magnetic components. Au and Ag have superior plasmonic properties than other metals, like Pt and Pd. Moreover, magnetic metals, such as Fe, Co, and especially Ni, can also exhibit some plasmonic properties although are quite dampened when compared to noble metals. Individual nanoparticles of noble metals, such as Au, Ag, Pt, Pd, and Cu demonstrated appropriate plasmon absorption intensity with PRA peaks at wavelengths between 300 to 520 nm [69]. On the other hand, the plasmonic properties of most magnetic components, in particular nonmetallic particles, are insignificant, but heterodimers showed enhanced plasmonic features when compared to individual noble metals particles: Plasmon absorption intensity of FePt-Au and Fe_3_O_4_-Au heterodimers increased up to three times in comparison with Au nanoparticles [81,89,90,109,158].

The plasmon absorption intensity is considered as one criterion for particles efficiency in related applications. For instance, in imaging and photothermal therapy applications, higher absorption intensity results in more efficient contrast and heat generation, respectively. Moreover, in all cases, PRA peaks were faced to a red-shift so that e.g., the peak of Au nanoparticles was shifted to 524–600 nm in heterodimers, while it is usually positioned at a wavelength of 520 nm. In fact, PRA peaks of uncombined plasmonic particles are commonly positioned at the range of UV and visible light, which show a minor performance for in vitro applications, since the penetration coefficient in this range of light into tissue is very low and is mostly absorbed in the skin layers (approximate penetration depth of UV: 30–100 µm, visible light: 40–250 µm). The red-shift moves PRA to NIR range with enhanced efficiency due to less absorbance [193,194,195].

Improved plasmonic properties of other noble metals were frequently reported in different heterodimers, such as Fe-Pt [85], Fe_3_O_4_-Ag [111,186], and Ni-Au [95] hybrids. The change of LSPR intensity and position of heterodimers was referred to electron transport across the interface of magnetic and plasmonic particles [90,196]. Then again, in most reported heterodimers, the LSPR sharpness was lower than that of single plasmonic components and the associated peaks were broadened and/or sporadically vanished [105,111]. A handful works reported the reduction of PRA intensity as well as blue-shifted positions for heterodimers [81,105,186]. Position (red/blue shift) and amplitude of localized plasmonic resonance closely depend on surface electronic properties of components, such as electron density, charge state, and electron exchange across the interface of nanoparticles. Increased free surface of plasmonic particles and their interface with other phases led to a change of energy levels, enhanced path range of surface plasmon resonance, and stronger electron exchange and larger red-shift [89]. Accordingly, magnetic-plasmonic heterodimers demonstrated significant red-shift with respect to pure plasmonic particles. Since the magnetic nanoparticles were the electron deficient components as compared to plasmonic particles, the combined heterodimers indicated lower surface plasmon resonance energy and decreased associated amplitude [89]. Furthermore, the joined magnetic nanoparticles could act as scattering centers that changed electronic band-band transition states of plasmonic structures and reduced resonance energy.

In the UV-Vis spectra of synthesized CoFe_2_O_4_-Pd heterodimers with randomly decorated form, absorption at all desired wavelengths of UV, visible, and especially NIR light was significantly enhanced when compared to individual CoFe_2_O_4_ nanoparticles, which improved their efficiency in associated biomedical applications, in particular, photothermal therapy [120]. In addition, in compliance with reported results in literature, there is no highlighted LSPR peak for Pd components in measured spectra at the NIR wavelengths.

QDs and oxide phases, like ZnO particles, are the other class of plasmonic materials that are reported. Heterodimers with QDs and oxidic plasmonic materials, like FePt-CdS [124], FePt/Fe_3_O_4_-CdSe [138], and CoFe_2_O_4_-ZnO [137] structures, typically presented lower PRA intensities as compared to bare plasmonic particles, although the final properties were sufficient for effective uses.

### 4.3. Electronic Structure

Magnetic and plasmonic properties of heterodimers can be attributed to the electronic structure of components. In general, the electronic structure depends on crystalline order, cations distribution, and oxidation states. These characteristics are commonly analyzed using techniques based on the X-ray sources such as X-ray diffraction (XRD), X-ray photoelectron spectroscopy (XPS), and X-ray absorption spectroscopy (XAS). In many works, XRD analyses were carried out to identify the phase and crystal structures of heterodimers components [197,198]. However, since in most heterodimers the quantity of guest part is very low (<5%), XRD results are inadequate. There are several reports on the investigation of structural parameters, like cations oxidation state and crystallite size, by using Rietveld structure refinement of XRD results [199]. For instance, Tremel’s research group analyzed XRD results of super-lattices of Fe_2_O_3_-Pd heterodimers via Rietveld refinement and found that iron cations appeared in both states of Fe^2+^ and Fe^3+^ and their ratio depended on the conditions of palladium nanoparticles [200].

For an accurate evaluation of chemical composition and electronic states at the surface of heterodimers, the XPS technique is commonly employed. By the identification of binding energies in XPS spectra, it was possible e.g., to prove the metallic states of Au in CoFe_2_O_4_-Au nanohybrids [201] or metallic Pd states in Fe_3_O_4_-Pd heterodimers [202].

A possibility to probe both electronic structure and crystallographic structure is offered by XAS. While the X-ray absorption near edge structure (XANES) contains information e.g., about oxidation state of the absorbing element and hybridization effects, the extended X-ray absorption fine structure (EXAFS) can be used to analyze distance, number, and type of neighboring atoms, as well as the coordination symmetry [203]. In contrast to diffraction methods, EXAFS analysis can be performed for both crystalline and amorphous materials.

For instance, Muraca and Siervo confirmed the formation of Ag-Fe_3_O_4_ (Ag: host) heterodimers using XANES measurements at the Fe-K edge [190]. Based on XANES and XMCD (Fe-L_3,2_ edges), they concluded that the Fe_3_O_4_ structure was composed of magnetite (Fe_3_O_4_) and maghemite (γ-Fe_2_O_3_) phases. The deviation from ideal Fe_3_O_4_ stoichiometry could be reduced by enhancing the Ag concentration. Furthermore, these analyses could be carried out to study the oxidation catalyst activity of structures. Najafishirtari et al. investigated the oxidation states of Fe (K edge), Cu (K edge), and Au (L_3,2_ edges) in Fe_3_O_4_-Au/Cu heterodimers and identified the dominant phase types and electronic structure modes before and after the oxidation process at different temperatures [204]. The utilization of XANES measurements to evaluate trends in variation of the oxidation states of heterodimers as a function of temperature was reported in some other works as well [205]. In the case of FePt-Fe_3_O_4_ heterodimers, a change of the Fe oxidation state was confirmed by an energy shift of XANES at the Fe-K edge towards lower energies with increasing temperature [206]. Pinader et al. evaluated the electrical structure of Fe_3_O_4_-Au heterodimers by the use of XANES and XMCD (Fe K and Au L_3,2_ edges) measurements and realized that the presence of Au nanoparticles led to a shift of Fe (K edge) spectra towards higher energies as a result of Fe oxidation state variation [207]. Moreover, the calculated results based on XANES and XMCD measurements revealed paramagnetic interactions of Au and magnetic particles, which originated from the diffusion of iron cations into the Au seeds structure, and the local creation of Au_x_Fe_y_ alloys [207]. Fe_3_O_4_-Ag heterodimers showed a similar trend of Fe K edge shifting that was related to different oxidation states because of surface effects [178]. Furthermore, based on the electronic structure investigation of CoFe_2_O_4_-Pd heterodimers using XANES measurements, we found that Pd decoration remarkably changed electronic structures, like cations, disordering in crystal structure of CoFe_2_O_4_ nanoparticles, which is closely connected to altered magnetic characteristics [120]. Even without further analysis, it can be seen in the spectra (Figure 8) that there is a small energy shift and changes in the characteristic fine structure, particularly for the Co ions. To connect the spectral features to changes in the electronic structure, a comparison to calculated spectra is envisaged. Moreover, electron energy loss spectroscopy (EELS) is a very useful technique for the elementary identification of electronic structure, composition, and oxidation state of nanoparticles at atomic scale. For instance, Fantechi et al. investigated the elemental composition and homogeneity of the two magnetic and plasmonic phases [105]. The results confirmed the homogeneous composition of iron oxide counterpart with no changes in the oxidation state. Additionally, fine structure analysis of EELS spectra proved the formation of magnetite phase, Fe_3_O_4_, [105]. Torruella et al. reported the three-dimensional (3D) visualization of oxidation state in several spinel structure core-shell heterodimers, such as FeO, Fe_3_O_4_, and Mn_3_O_4_ structures using EELS measurements [208,209]. The direct mapping of Mn^2+^, Mn^3+^, Fe^2+^, and Fe^3+^ cations distribution in the core and shell crystalline lattice of nanoparticles was reconstructed in three dimensions with atomic resolution, which provided significant improvement towards a precise understanding the correlation of the electronic and functional properties of heterodimers [208,209].

In summary, the combination of components to heterodimers considerably changes various parameters of the electronic structures, which directly affect other features. Thus, a detailed investigation of electronic structures can be very helpful. Some characteristics, like cations disordering and crystallinity, need further, more focused studies.

### 4.4. Biocompatibility and Physiochemical Stability

In order to successfully utilize various nanomaterials in biomedical applications, physiochemical stability, in particular, biocompatibility, are essential factors. Biocompatibility of heterodimers should be in the certain level with minimum side effects and appropriate performance for both in vitro and in vivo mediums. In hybrid structures, the biocompatibility of both magnetic and plasmonic components should be considered.

The biocompatibility of nanoparticles was evaluated by using in vitro analyses, animal model testing, and finally clinical trials before the utilization in biomedical applications. Most studies were carried out through in vitro analyses such as cell viability, toxicity, and MTT assays. Noble metals as an ideal choice for plasmonic part indicated excellent biocompatibility that improved the features of combined structures. Particularly, Au and Ag nanoparticles for advanced biocompatibility, anticancer, and antibacterial properties of magnetic-plasmonic heterodimers were frequently used [108,109,111,210,211,212]. This affirmative role is extra-decisive in the case of more toxic magnetic components, like CoFe_2_O_4_ particles. For instance, Kooti et al. found antibacterial as well as enhanced biocompatibility in CoFe_2_O_4_-Ag hybrids [115]. Recently, Chen et al. reported using polymeric coating on Fe_3_O_4_-Au hybrids to improve the biological stability and loading efficiency of doxorubicin anticancer drug [109]. Applying a biocompatible layer on the heterodimers surface is useful to decrease toxicity, although in most structures, the magnetic and plasmonic properties are dramatically weakened. Schick et al. controlled the biocompatibility of MnO-Au heterodimers through partially silica coating [121].

Nonmetallic plasmonic materials showed two opposite approaches: While oxide structures like ZnO nanoparticles had suitable biocompatibility and antibacterial behavior, most QDs, like CdS and CdSe, were toxic and faced practical limitations [213]. Regardless of electronic nature (metal/nonmetal), magnetic ingredients commonly demonstrated acceptable biocompatibility and some of them, like Fe_3_O_4_ nanoparticles, were already approved for biomedical applications [95].

Physicochemical stability is one of the most important characteristics of heterodimers. The improvement of this factor is more highlighted in metallic-metallic hybrids, since the metallic magnetic and especially plasmonic particles have a strong tendency to agglomeration because of some specific physical-chemical properties, like surface to volume ratio [214]. Combined structures of host-guest pattern could increase chemical stability and monodispersity. Physicochemical stability of nanoparticles does not only prevent phase transformations, but it also assists in the easier control of magnetic and particularly plasmonic features. Chemically stable noble metals could be used to protect magnetic particles from oxidation and changes of their electronic states, which instantly affect practical properties [85]. Table 1 indicates the effectiveness trends of different heterodimers on the biocompatibility and magnetic-plasmonic characteristics.

**Summary** **4.** *The combination of magnetic and plasmonic nanoparticles to heterodimer structures significantly affects magnetic and plasmonic properties as well as biocompatibility and physicochemical stability and can be used to tailor the characteristics for various applications. The electronic structure has turned out to play a key role in understanding the physics behind.*

## 5. Improved Performances

The combination of magnetic and plasmonic components does not only present various properties in a single structure, but also causes to emerge new applications. The performances of heterodimers in above-mentioned applications depend on structure nature, surface modifications, and particles size and morphology, so that some ideal choices could be introduced for each application. Selected examples of heterodimers used in specific applications are reviewed in the following.

### 5.1. Magnetic Hyperthermia

**Figure 9:**
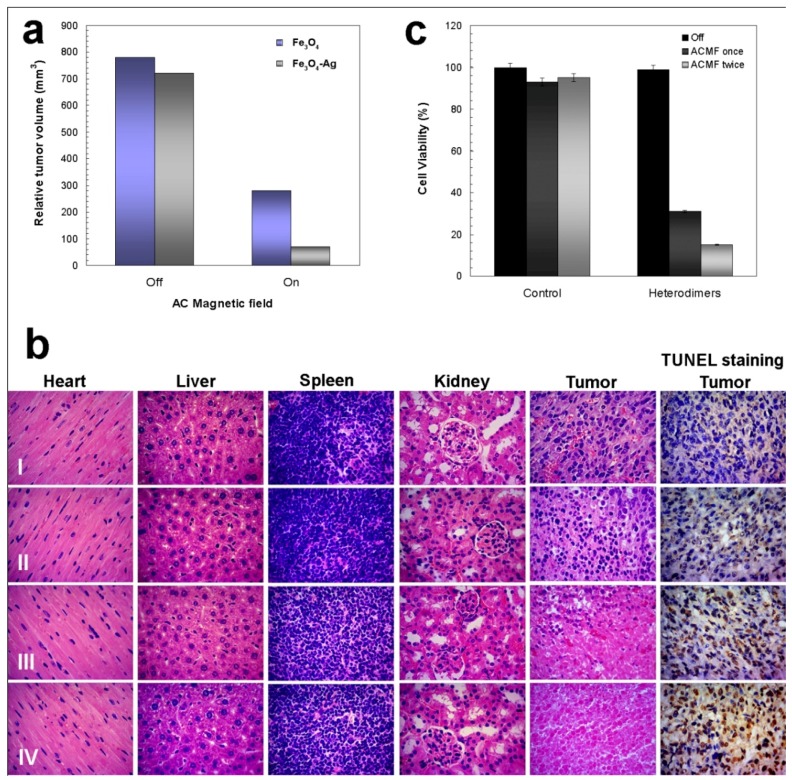
Therapeutic effects of heterodimers during magnetic hyperthermia in vivo antitumor effect of subcutaneous SMMC-7721 tumor in mice after seven-day treatment period by Fe_3_O_4_-Ag heterodimers (**a**). Data taken from reference [111]. Histological analysis of mouse organs and tumor on the seven-day after treatments for (I): Fe_3_O_4_ nanoparticles without ACMF (II): Fe_3_O_4_-Ag heterodimers without ACMF (III): Fe_3_O_4_ nanoparticles with ACMF, and (IV): Fe_3_O_4_-Ag heterodimers with ACMF (**b**). Reproduced with permission from [111], copyright (2017) Elsevier. Cell viability of the 4T1 cells after treatments by Fe_3_O_4_-GQD heterodimers (**c**). Data taken from reference [142].

Although the utilization of magnetic-plasmonic heterodimers in hyperthermia is relatively new, it has been reported in several works [215,216,217,218]. Fe_3_O_4_ and other biocompatible ferrites nanoparticles turned out to be perfect candidates for hyperthermia applications [219,220]. However, their insufficient heat transferring coefficients decreased the efficiency of the treatment. Accordingly, the positive role of noble metals in hybrid structures was reported to enhance cancerous cells thermosensitivity and death under in vitro and in vivo hyperthermia treatments [221,222]. As mentioned in Section 4.1, most para-/diamagnetic noble metals reduced the saturation magnetization and magnetic susceptibility, but their significant effects on the heat transferring could ameliorate the hyperthermia efficiency in total. Gu’s research group recently studied the magnetic hyperthermia behavior of Fe_3_O_4_-Ag heterodimers for in vitro and in vivo models [111]. Hyperthermia treatments prevented the rapid growth of SMMC-7721 tumor volume in mice samples and the prevention efficiency of heterodimers was much higher than that of pure Fe_3_O_4_ nanoparticles. In fact, hyperthermia treatments by using heterodimers led to a full stop of the tumor growth over a seven-day period (Figure 9a). In addition, the histopathology results of samples proofed the excellent efficiency of heterodimers hyperthermia, which revealed massive necrosis and fibrosis symptoms of cancerous tissues (Figure 9b). TUNEL assay results demonstrated well the apoptosis of cancerous cells (brown cells) in the heterodimers sample. In another work, the hyperthermia performance of Fe_3_O_4_-graphene QDs heterodimers was studied by Yao et al. [138]. Those heterodimers generated sufficient magnetic heating under the applied AC magnetic field to increase in vitro death of 4T1 cancerous cells when compared to control samples (Figure 9c).

Moreover, we observed that synthesized CoFe_2_O_4_-Pd heterodimers with different fluid concentrations and particles sizes generated more heat than individual CoFe_2_O_4_ nanoparticles in an applied AC magnetic field, which led to enhanced efficiency in magnetic hyperthermia applications [120]. In addition, calculated SAR values for ferrofluids with the same volume and concentration increased from 38 W/g_tot_ for CoFe_2_O_4_ nanoparticles (with mean hydrodynamic diameter of 24 nm) to 50 W/g_tot_ for CoFe_2_O_4_-Pd heterodimers as a result of magnetic anisotropy enhancement of heterodimer structures.

In addition to common intra-tumor magnetic hyperthermia, intra-cellular treatments have attracted much attention in recent years. Intra-cellular hyperthermia demonstrated an effective therapeutic process through selective penetration into the cancerous cells and lethal heat generation from inside the cell [223,224]. Fortin et al. investigated the performance of different iron oxide phases, like maghemite (γ-Fe_2_O_3_) and cobalt ferrite (CoFe_2_O_4_), as nanoheaters for intra-cellular hyperthermia in human prostatic adenocarcinoma cells (PC3) [225]. They found that the magnetic heating efficiency at an intra-cellular scale is lower than in solution for samples with the same magnetic elements concentrations, because the dominant heating mechanism of intra-cellular hyperthermia was Neél relaxation, while the Brownian relaxation mechanism was suppressed in intra-cellular models due to the immobilization of nanoparticles [66]. Moreover, Di Corato et al. employed different types of magnetic nanomaterials, such as maghemite (nanospheres, nanocubes, and nanoflowers), liposomal encapsulated maghemite, cobalt ferrite, and γ-Fe_2_O_3_-Au heterodimers to investigate magnetic hyperthermia efficiency in the cellular environment [226]. The achieved results confirmed the complete inhibition of the Brownian loss mechanism in cellular conditions [226]. Ota et al. evaluated the performance of Fe_3_O_4_ nanoparticles in intra-cellular hyperthermia by employing three formats of aqueous solution, epoxy mounted, and cellular samples under AC magnetic field using a theoretical model of measured AC hysteresis loops [227]. They reported that, in the cellular conditions, the dipole-dipole and particle-cell interactions inhibited the magnetic moments and particles rotations, respectively, yielding lower heat dissipation in the cellular environment as compared to solution and mounted samples [227].

### 5.2. Photothermal Therapy

As an advanced mode of active photodynamic treatment, photothermal therapy was applied to obliterate tumor cells through locally generated heat by plasmonic nanoparticles upon irradiation with photons of certain wavelength and energy [69,228]. Noble metals are the main class of plasmonic materials, which locally generate heat that is based on SPR phenomenon activated by UV, visible, and NIR waves. Heterodimers composed of Au nanoparticles were most frequently used to rise temperature up to around 43 °C and destroy cancerous cells under NIR radiation [229,230]. In addition to common Au and Ag nanoparticles, the use of Pt, Pd, and Cu metallic particles in PTT applications was reported [231,232,233]. For example, Ding et al. realized the enhanced heating efficiency of Fe_3_O_4_-Pt heterodimers during simultaneous PTT and radiation therapy processes [167].

As mentioned in Section 4.2, some of the premier advantages of heterodimers when compared to individual plasmonic components are the red-shift of LSPR peaks as well as increased photon energy absorption and localized heating. When considering the safety and suitable penetration ability of NIR beam, the most practical uses of plasmonic nanoparticles for photothermal therapy were carried out at NIR range. The first and second absorption windows of NIR laser were determined at wavelengths of 800–820 nm and 1450–1470 nm, respectively, which considerably activated photothermal heating [143]. In photothermal therapy, NIR biological windows refer to two regions of wavelengths with maximum penetration depth in tissue, which are located between 650–950 nm (first window), with the optimal value at about 808 nm and 1000–1350 nm (second window), and with an optimal value at about 1064 nm [234,235]. The limits for these NIR biological windows are mainly determined by the absorption coefficients of blood and water. In detail, NIR photons are absorbed by two hemoglobin types of blood at short wavelengths (410–600 nm), i.e., before the first window, as well as by the water components of human tissue at long wavelengths. The NIR transparency at wavelengths in the biological windows originates from both low absorption and low scattering [234]. The latter is the main interaction between light and tissue in the biological windows.

**Figure 10:**
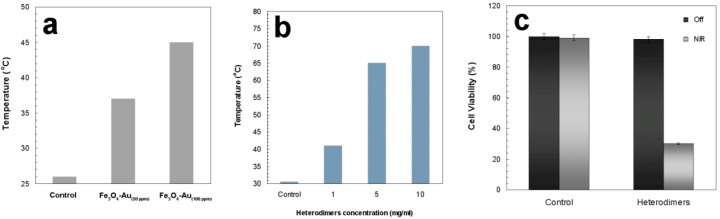
Therapeutic photothermal effects of heterodimers. Photothermal heating of Fe_3_O_4_-Au heterodimers with different Au concentrations under 808 nm near infra-red (NIR) radiation for 300 s (**a**). Data taken from reference [158]. Photothermal heating of Fe_3_O_4_-GQD heterodimers under 808 nm NIR radiation for 300 s (**b**). Data taken from reference [142]. Cell viability of the 4T1 cells with treatment by Fe_3_O_4_-GQD heterodimers under NIR radiation for 3 min (**c**). Data taken from reference [142].

Although some noble metals, like Pd particles, present no LSPR peaks in these ranges, they were efficiently utilized for photothermal therapy under NIR radiation with a wavelength of 808 nm, owing to the absorption enhancement of heterodimers [236]. The temperature of nanoparticles increased from an ambient level (25 °C) to the therapeutic temperature of cancerous cells (43 °C) in an acceptable time period (300 s). A desirable rate of temperature rising under NIR radiation with a wavelength of 808 nm was reported for Fe_3_O_4_-Au and Fe_3_O_4_-graphene QDs heterodimers (Figure 10a,b) [142,158]. Furthermore, in vitro analyses illustrated the destruction of 4T1 cancerous cells up to 70% under NIR irradiation for three minutes, which confirmed the promising performance of magnetic-plasmonic heterodimers in photothermal therapy applications (Figure 10c) [142].

Most of the studies carried out on the field of magnetic-plasmonic heterodimers focused on the separate investigation of magnetic and optical hyperthermia treatments, but recently some works introduced the amplification effects of heating efficiency under simultaneously applied AC magnetic fields and NIR lasers. For instance, Espinosa et al. reported that iron oxide nanocubes exposure to both AC magnetic field and NIR laser irradiation (808 nm) demonstrated a 2- to 5-fold enhancement in heating efficiency when compared to individual magnetic stimulation [237]. In addition, they found that in solid tumors in vivo, separate single treatments (magnetic hyperthermia or laser irradiation) reduced tumor growth while simultaneous processes led to complete tumor regression [237]. In another work, Das et al. synthesized Fe_3_O_4_-Ag heterodimer nanoflowers with boosted SAR value more than one order of magnitude under a simultaneously applied AC magnetic field and NIR laser irradiation for synergistic magnetic and photothermal therapies [238]. Recently, Ovejero et al. presented considerable enhancement in the treatment effectiveness of iron oxide-Au nanorods excited by infrared beam and alternating magnetic field [239].

### 5.3. Bioimaging

Nowadays, bioimaging and biosensing play critical roles in diagnosis, so that without these tools, diagnostic and therapeutic processes of many diseases are completely disturbed. These techniques could be applied for both in vitro and in vivo applications. While biosensing was mostly used in vitro, bioimaging methods were very helpful for in vivo cases. Biosensing focused on monitoring of biologic agents, toxic compounds, drugs, and therapeutic substances [240]. Since biosensing that is typically based on measurements of colorimetry, plasmon resonance, UV-Vis absorption, fluorescence emission, and solution phase dielectric constant, the noble metals were introduced as the ideal choices for this aim and Au-containing compounds were the most common biosensing structures [241,242,243]. Moreover, Fe_3_O_4_-Ag [186], FePt/Fe_3_O_4_-CdSe [156], and Fe_3_O_4_-ZnS [244] heterodimers were employed for biosensing uses. Demeritte et al. successfully designed Fe_3_O_4_-Au/graphene nanohybrids as a platform of Alzheimer diagnosis biomarkers [212].

**Figure 11:**
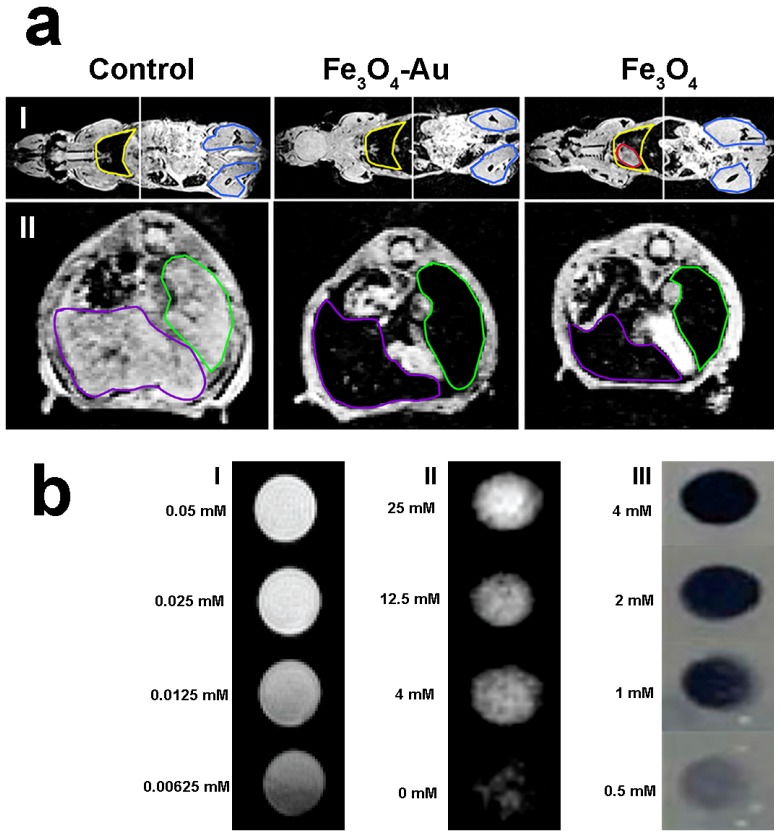
Performance of heterodimers in bioimaging applications. MRI images of the mouse tissues 24 h after injection of Fe_3_O_4_ and Fe_3_O_4_-Au contrast agents from two sections, (**I**) first section scan of ling (yellow), muscle (blue), and heart (red), (**II**) sectional plane (white stripe in (**I**)) of liver (purple) and spleen (green). Reproduced with permission from [108], copyright (2015) Elsevier (**a**), Multimodal imaging with contrast agents of Fe_3_O_4_-Au heterodimers with different concentrations for (I): MRI, (II): CT, and (III): PA techniques (**b**). Reproduced with permission from [109], copyright (2017) Royal Society of Chemistry.

The efficiency of most imaging techniques, such as MRI, CT, PET, ultrasonic (US), and NIR optical imaging (OI), is affected by magnetic and/or plasmonic properties of nanomaterials as contrast agents [245]. Landgraf et al. investigated the performance of Fe_3_O_4_-Au heterodimers as contrast agents in animal models MRI applications and confirmed their excellent effectiveness (Figure 11a) [108]. In addition, Reguera et al. realized the simultaneous improvement of MRI, CT, and PA techniques by using Fe_3_O_4_-Au contrast agents (Figure 11b) [109].

One of the most promising imaging modalities is the magnetic particle imaging (MPI), which can be used for diagnosis and therapeutic applications, such as cardiac imaging, solid tumors screening imaging, and cell tracking. More details about this technique can be found in the work of Gleich and Weizenecker [246], who introduced the first concept of MPI for obtaining high-resolution images by tracing the signal from magnetic nanoparticles in oscillating magnetic fields applied. While in MRI, the influence of magnetic contrast agents on the relaxation of surrounding tissue is analyzed; MPI directly maps the 3D distribution of the magnetic component. MPI can be as widely employed as MRI, even with less side effects. Main advantages of MPI are high resolution (around 0.5 mm), high contrast, no radiation, no iodination, and fast imaging process [247]. Goodwill et al. presented safe medical MPI for chronic kidney disease (CKD) patients using superparamagnetic iron oxide nanoparticles (SPIONs) tracers [247]. The used nanoparticles revealed significantly enhanced contrast, sensitivity, and resolution of imaging (up to 250 µm), in comparison with X-ray and CT iodinated angiography, particularly for CKD patients. Arami et al. conjugated two different types of poly-ethylene-glycol to monodisperse carboxylated iron oxide nanoparticles labeled with near infrared fluorescent molecule for in vitro and in vivo MPI applications [248]. They confirmed the tracers’ biodistribution in liver and spleen by MPI signals and found that the signals intensity of MPI tracers in the blood strongly depended on their plasmatic clearance pharmacokinetics [248]. In another work, Ota et al. comprised the performance of magnetic nanoparticles as a blood-pooling agent and commercial tracer agent for MPI applications and found that particles size, structure, and distribution were the most efficient parameters on the harmonic intensity of MPI signals [249]. Recently, Song et al. presented interesting results about the tailoring of Janus nanoparticles that are composed of iron oxide and semiconducting polymer phases for MPI and fluorescence imaging [250]. The utilized heterodimers introduced the MPI signal intensity up to three times of commercial MPI contrast (Vivotrax) and seven times of MRI contrast (Feraheme) for the same iron concentration [250].

As a result, MPI techniques were presented as a new promising imaging modality for a wide range of future biomedical.

### 5.4. Drug Delivery

As a one of new pharmaceutical approaches, drug delivery increases drug effectiveness on its targeted organ with minimal side effects. Currently, EPR is the most common delivery mechanism. It is confronted with some disadvantages, such as insufficient rate of drug delivery and release [159]. Heterodimer structures could be potentially used to overcome these inadequacies. Magnetic targeting and thermal-releasing of therapeutic agents were activated through magnetic components [72]. For instance, Fe_3_O_4_-graphene QDs heterodimers as the nanocarriers of anti-cancer doxorubicin molecules led to an enhanced drug release of up to 30% in comparison with EPR mechanism [142].

Moreover, noble metals, especially Au nanoparticles, were considered as stable nanocarriers due to their significant surface functionability [251]. In addition, these particles could activate thermal- and photo-releasing mechanisms through exposure to electromagnetic radiations for therapeutic applications, like cancer and HIV treatments [252,253]. In fact, these structures acted as NIR-sensitive platforms, while the surface bonding was loosed under irradiation and causes drug release with acceptable rate [254,255].

**Summary** **5.** *Tailored magnetic-plasmonic heterodimers can be employed e.g., for hyperthermia with enhanced efficiency, as consummate contrast agents for multimodal imaging and show considerable performance in drug delivery uses.*

## 6. Conclusions and Future Perspective

This review aimed to briefly present fascinating recent achievements in the area of magnetic-plasmonic heterodimer nanostructures for biomedical diagnosis and treatments focusing on the less-known characteristics of these nanoparticles, including type and interfacial morphology of designed structures, specific features, and emerging applications. Various heterodimers composed of plasmonic components of noble metals and quantum dots and magnetic ingredients, like metallic superparamagnetic nanoparticles and ferrite compounds, have been increasingly employed for many biomedical applications, such as magnetic hyperthermia, photothermal therapy, bioimaging, and drug delivery. The main benefit of heterodimers is the synergistic gain of magnetic and plasmonic properties in unique structures with mostly enhanced performance when compared to individual components. Despite many reported works on the magnetic and plasmonic features of heterodimers, a lack of certain fundamental investigations of their mutual correlations has been identified. In order to design new heterostructures with optimum performance, the electronic structure should be investigated in more detail, since it is probably the most important property defining the mutual influence of the two constituents by electronic hybridization effects and exchange interactions. For experimental works, element-specific methods, like x-ray absorption spectroscopy, seem to be a powerful method to monitor even subtle changes.

To identify novel promising candidates and understand the mutual correlations in more detail, theoretical support is crucial. Substantial improvements in supercomputer architecture and code development over the last decade point towards a feasible treatment of nanoscale metal/oxide interfaces and surfaces. For the known biocompatible heterodimers also exhibiting significantly enhanced physiochemical stability, further in vivo studies are needed to make use of the synergistic features of heterodimers in biomedical theranostics in the future.

In conclusion, the first steps in the development of heterodimers, as a new generation of multipurpose nanomaterials, have already been made. For a successful continuation of this promising research field and establishing this class of materials in applications, interdisciplinary cooperation is vital including e.g., chemistry, material sciences, theoretical and experimental physics, biology, and medicine.

## Figures and Tables

**Table 1:** Efficacies of different heterodimers on magnetic-plasmonic characteristics like saturation magnetization M_S_, remanent magnetization M_r_, magnetic coercivity field H_C_, effective anisotropy constant K, blocking temperature T_B_, electro-magnetic absorption amplitude as well as shift of localized surface plasmon resonance (LSPR) peak and biocompatibility.

Composition	Magnetic Characteristics					Plasmonic Characteristics		Biocompatib.	Ref.
	M_S_	M_r_	H_C_	K	T_B_	Abs. ampl.	LSPR Shift		
FePt-Au	n/a	n/a	n/a	n/a	n/a	+	Red	n/a	[89]
FePt-Au	-	-	=	-	-	+	Red	n/a	[90]
FePt-CdS	-	n/a	n/a	n/a	n/a	-	Red	n/a	[123]
Fe-Au	- =	- =	=	n/a	n/a	-	Red	n/a	[81]
Fe-Pd	-	n/a	n/a	n/a	n/a	+	Red	n/a	[82]
Ni-Au	n/a	n/a	n/a	n/a	n/a	+	n/a	n/a	[83]
Fe_3_O_4_-Au	n/a	n/a	n/a	n/a	n/a	+	Red	n/a	[81]
Fe_3_O_4_-Au	-	-	-	n/a	n/a	-	Red	n/a	[105]
Fe_3_O_4_-Au	-	n/a	n/a	n/a	n/a	+	n/a	+	[158]
Fe_3_O_4_-Au	-	n/a	n/a	n/a	+	+	Red	+	[109]
Fe_3_O_4_-Ag	- =	n/a	n/a	n/a	n/a	+	Red	+	[111]
Fe_3_O_4_-Ag	- =	- =	=	n/a	n/a	+	Blue	+	[186]
Fe_2_O_3_-Ag	n/a	n/a	n/a	n/a	n/a	+	Red	+	[190]
MnFe_2_O_4_-Ag	-	-	-	n/a	n/a	n/a	n/a	n/a	[94]
CoFe_2_O_4_-Ag	-	-	-	n/a	n/a	n/a	n/a	+	[115]
CoFe_2_O_4_-ZnO	-	-	-	n/a	n/a	-	Red	n/a	[137]
Fe_3_O_4_-Pd	=	=	=	n/a	n/a	n/a	n/a	n/a	[113]
Fe_3_O_4_-antibody dye	=	n/a	n/a	n/a	n/a	+	Red	+	[143]
Fe_3_O_4_-GQDs@SiO_2_	-	n/a	n/a	n/a	n/a	+	n/a	+	[138]
FePt/Fe_3_O_4_-CdSe/SiO_2_	-	-	n/a	n/a	n/a	-	Red	n/a	[156]
MnO-Au	-	-	-	n/a	n/a	n/a	n/a	n/a	[121]

Abbreviations: (+): increase (-): decrease (- =): slight decrease (=): no change, and (n/a): not available.

## Abbreviations

CKD	Chronic kidney disease
CL	Colloidal lithography
CT	Computed tomography
EELS	Electron energy loss spectroscopy
EPR	Enhanced permeability and retention
EXAFS	Extended X-ray absorption fine structure
FDA	US food and drug administration
H_C_	Magnetic coercivity
HIV	Human immunodeficiency virus/AIDS
HRTEM	High resolution transmission electron microscopy
K	Magnetic anisotropy
LSPR	Localized surface plasmon resonance
M_r_	Remanent magnetization
M_S_	Saturation magnetization
MFH	Magnetic fluid hyperthermia
MRI	Magnetic resonance imaging
MPI	Magnetic particle imaging
NIR	Near infrared
NPs	Nanoparticles
OI	Near infrared optical imaging
OM	Optical microscopy
PA	Photoacoustic imaging
PEG	Polyethylene glycol
PET	Positron emission tomography
PTT	Photothermal therapy
PVP	Polyvinylpyrolidone
QDs	Quantum dots
SAR	Specific absorption rate
SPA	Surface plasmon absorption
SPIONs	Superparamagnetic iron oxide nanoparticles
SQUID	Superconducting quantum interference device
T_B_	Blocking temperature
TEM	Transmission electron microscopy
TUNEL	Terminal deoxynucleotidyl transferase dUTP nick end labeling
US	Ultrasonic
UV	Ultraviolet
UV-Vis	Ultraviolet-visible
VSM	Vibrating sample magnetometer
XANES	X-ray absorption near edge structure
XAS	X-ray absorption spectroscopy
XMCD	X-ray magnetic circular dichroism
XPS	X-ray photoelectron spectroscopy
XRD	X-ray diffraction
